# Stoichiometric Conversion of Maltose for Biomanufacturing by *In Vitro* Synthetic Enzymatic Biosystems

**DOI:** 10.34133/2022/9806749

**Published:** 2022-07-01

**Authors:** Guowei Li, Xinlei Wei, Ranran Wu, Wei Zhou, Yunjie Li, Zhiguang Zhu, Chun You

**Affiliations:** ^1^Tianjin Institute of Industrial Biotechnology, Chinese Academy of Sciences, 32 West 7th Avenue, Tianjin Airport Economic Area, Tianjin 300308, China; ^2^College of Biotechnology, Tianjin University of Science and Technology, 1038 Dagu Nanlu, Hexi District, Tianjin 300457, China; ^3^National Technology Innovation Center of Synthetic Biology, Tianjin 300308China

## Abstract

Maltose is a natural *α*-(1,4)-linked disaccharide with wide applications in food industries and microbial fermentation. However, maltose has scarcely been used for *in vitro* biosynthesis, possibly because its phosphorylation by maltose phosphorylase (MP) yields *β*-glucose 1-phosphate (*β*-G1P) that cannot be utilized by *α*-phosphoglucomutase (*α*-PGM) commonly found in *in vitro* synthetic enzymatic biosystems previously constructed by our group. Herein, we designed an *in vitro* synthetic enzymatic reaction module comprised of MP, *β*-phosphoglucomutase (*β*-PGM), and polyphosphate glucokinase (PPGK) for the stoichiometric conversion of each maltose molecule to two glucose 6-phosphate (G6P) molecules. Based on this synthetic module, we further constructed two *in vitro* synthetic biosystems to produce bioelectricity and fructose 1,6-diphosphate (FDP), respectively. The 14-enzyme biobattery achieved a Faraday efficiency of 96.4% and a maximal power density of 0.6 mW/cm^2^, whereas the 5-enzyme *in vitro* FDP-producing biosystem yielded 187.0 mM FDP from 50 g/L (139 mM) maltose by adopting a fed-batch substrate feeding strategy. Our study not only suggests new application scenarios for maltose but also provides novel strategies for the high-efficient production of bioelectricity and value-added biochemicals.

## 1. Introduction

*In vitro* synthetic enzymatic biosystems are cell-free systems comprised of three or more enzymes in one pot to implement complicated biochemical reactions [1]. This emerging biosystem for biomanufacturing features several advantages over *in vivo* systems (microbial fermentation), including fewer side reactions and easier adjustment of reaction conditions [2] and hence often result in high product yields and fast reaction rates [3–6]. Carbohydrates such as glucose, sucrose, cellobiose, cellulose, and starch are promising substrates for *in vitro* synthetic enzymatic biosystems to produce hydrogen [7–9], bioelectricity [10–12], and biochemicals such as *myo*-inositol [4, 5, 13], fructose 1,6-diphosphate (FDP) [14], D-allulose [6], glucosamine [15], polyhydroxybutyrate [16], and monoterpenes [17]. The first step of these *in vitro* biosystems is the phosphorylation of carbohydrate substrates to either glucose 1-phosphate (G1P) or glucose 6-phosphate (G6P), which are two important intermediates for biosynthesis. Phosphorylation of glucose through either the ATP-dependent hexokinase (HK) or polyphosphate glucokinase (PPGK) leads to the formation of G6P. Sucrose, cellobiose, cellulose, and starch are phosphorylated by corresponding phosphorylases to generate G1P, which can be further isomerized to G6P by phosphoglucomutase (PGM).

Maltose is a natural disaccharide consisting of two molecules of glucose joined by an *α*-(1,4) glycosidic linkage. As the key structural motif of starch, maltose can be readily produced from starch hydrolysis catalyzed by amylase [18–20]. The practicability and relatively low cost of maltose render this disaccharide a preferred material for the industrial production of confectioneries, ice creams, pastries, and beverages. Maltose has also been used as a carbon source for fermentation [21–23], as well as a substrate for whole-cell biocatalysis to produce trehalose [24]. Despite that maltose is one of the most readily available carbohydrates, few *in vitro* synthetic enzymatic biosystems have been reported to utilize maltose as the substrate for biomanufacturing. The main difference of maltose from the other abovementioned disaccharides and polysaccharides is that its phosphorylation catalyzed by maltose phosphorylase (MP) yields *β*-glucose 1-phosphate (*β*-G1P), whereas the phosphorylation of sucrose, cellobiose, cellulose, and starch yields *α*-G1P. Correspondingly, the PGMs used in our previously established *in vitro* biosystems were all *α*-phosphoglucomutases (*α*-PGMs) which is a key enzyme linking the glycolysis and gluconeogenesis pathways for the interconversion of *α*-G1P and *α*-/*β*-G6P [25, 26] and cannot convert *β*-G1P to *α*-/*β*-G6P [27]. Noted that the interconversion between *α*- and *β*-G6P occurs spontaneously, whereas the interconversion between *α*- and *β*-G1P does not. These facts lead to the mismatch between MP and *α*-PGM which might account for the lack of maltose-based *in vitro* biosystems to date.

In this study, two *in vitro* synthetic enzymatic biosystems were designed for the stoichiometric utilization of maltose for biomanufacturing. At first, a three-enzyme reaction module consisting of MP, *β*-phosphoglucomutase (*β*-PGM), and PPGK was designed for the stoichiometric conversion of maltose to G6P. Then, more downstream enzymes were added to prepare two *in vitro* synthetic enzymatic biosystems for the high-yield generation of bioelectricity and FDP, respectively. Our study not only provides new application scenarios for maltose but also suggests novel strategies for the high-efficient synthesis of other products such as hydrogen, inositol, glucosamine, and rare sugars in the future.

## 2. Materials and Methods

### 2.1. Experimental Design

**Figure 1:**
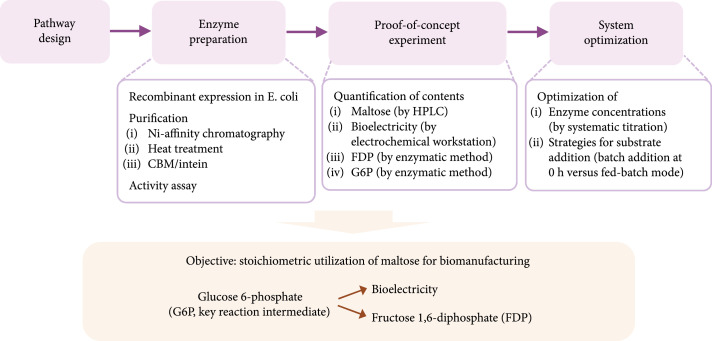
Schematic workflow of the experimental design.

Aiming at the stoichiometric utilization of maltose for biomanufacturing, we first designed the enzymatic pathways capable of converting both glucose units of maltose into G6P, which could be further utilized by other enzymes for biomanufacturing. Next, enzymes in the pathways were expressed and purified individually. Proof-of-concept experiments were conducted to evaluate the feasibility of our designed pathway. The consumption of maltose and the production of bioelectricity and FDP as well as the accumulation of G6P were quantified. If necessary, the reaction systems would subsequently be optimized to improve their product yields. The schematic workflow is illustrated in Figure 1.

### 2.2. Chemicals and Strains

Unless otherwise noted, chemicals used in this study were purchased from Sigma-Aldrich (St. Louis, MO, USA), Sinopharm (Shanghai, China), or Solarbio (Beijing, China). PrimeSTAR Max DNA Polymerase (Takara, Tokyo, Japan) was used for PCR. *Escherichia coli* TOP10 (CWBio, Beijing, China) and *E. coli* BL21 (DE3) (Invitrogen Co., Carlsbad, CA, USA) were used for DNA manipulation and recombinant protein expression, respectively.

### 2.3. Plasmid Construction

Plasmids pET20b-*mp* for the expression of MP from *Bacillus subtilis* strain 168 (UniProt entry number: O06993), pET20b-*βpgm* for the expression of *β*-PGM from *Pyrococcus horikoshii* OT3 (NCBI reference sequence: WP_010884842.1), and pET20b-*rpe* for the expression of ribulose 5-phosphate 3-epimerase (RPE) from *Thermotoga maritima* MSB8 (UniProt entry number: Q9X243) were constructed by simple cloning [28]. Primers for PCR were synthesized by Azenta Life Sciences (Suzhou, China), and their sequences were listed in Table S1. Plasmids for the expression of recombinant enzymes, including PPGK mutant 4-1 [29], glucose 6-phosphate dehydrogenase (G6PDH) mutant 4-1 [30], 6-phosphogluconate dehydrogenase (6PGDH) [31], diaphorase (DI) [32], transaldolase (TAL) [33], transketolase (TK) and fructose-bisphosphate aldolase (ALD) [7], ribose 5-phosphate isomerase (RPI) [34], triose phosphate isomerase (TIM) [31], pyrophosphate-dependent phosphofructokinase (PP_i_-PFK) [35], the fusion protein CBM-intein-phosphoglucose isomerase (PGI) [36], and the fusion protein CBM-intein-fructose bisphosphatase (FBP) [37], were constructed as previously described. The sequences of constructed plasmids were confirmed by DNA sequencing (Azenta Life Sciences, Suzhou, China).

### 2.4. Enzyme Preparation

*E. coli* BL21(DE3) transformed with the constructed plasmid was cultivated at 37°C in LB medium containing either 100 *μ*g/mL ampicillin or 50 *μ*g/mL kanamycin. Recombinant protein expression was induced by adding a final concentration of 0.01–0.1 mM of isopropyl-*β*-D-thiogalactopyranoside (IPTG) when the absorbance of the bacterial culture at 600 nm reached 0.8–1.2. The bacterial culture was further incubated at 37°C for 4 h or at 16°C for 20 h. The cells were harvested by centrifugation at 4°C, washed twice with 50 mM HEPES buffer (pH 7.0), and resuspended in 50 mM HEPES buffer (pH 7.5) containing 100 mM NaCl. The suspended cells were then placed in an ice bath and lysed by a Fisher Scientific Sonic Dismembrator Model 500 ultrasonicator. Three approaches, including nickel affinity chromatography with Ni Sepharose 6 Fast Flow medium (GE Healthcare, USA), heat precipitation at 70°C for 20 min, and affinity adsorption of carbohydrate-binding module (CBM) on regenerated amorphous cellulose followed by self-cleavage of intein [36, 37], were adopted for enzyme purification (Table S2). Each enzyme was expressed and purified individually. The purities of the recombinant enzymes were analyzed by SDS-PAGE. The concentrations of proteins were determined using the Bradford method with bovine serum albumin as standard.

### 2.5. Enzyme Activity Assay

The activity of *β*-PGM was determined based on the generation of G6P. Reactions were performed at 37°C in 100 mM HEPES (pH 7.0) containing 10 mM *β*-G1P and 10 mM MgCl_2_. The reaction was initiated by the addition of *β*-PGM and stopped by cooling in an ice-water bath. G6P was determined by mixing 50 *μ*L of the sample with 200 *μ*L of 100 mM HEPES buffer (pH 7.0) containing 10 mM MgCl_2_, 1 mM NAD^+^, and 1 U/mL G6PDH. After incubation at 37°C for 15 min, the increase of absorbance at 340 nm was measured. One enzyme unit was defined as the amount of enzyme that produced 1 *μ*mol of G6P from *β*-G1P per minute. Optimal temperature of *β*-PGM was determined in 100 mM HEPES (pH 7.0) within the temperature range of 25–90°C. Optimal pH of *β*-PGM was tested at 37°C in 100 mM Bis-Tris buffer (pH 6.0–7.0) and 100 mM HEPES buffer (pH 7.0–8.0).

The activity of MP was determined at 37°C according to the generation of *β*-G1P from maltose. Reactions were performed in 100 mM HEPES buffer (pH 7.0) containing 50 mM NaH_2_PO_4_-Na_2_HPO_4_ and 10 mM maltose. The reaction was initiated by the addition of MP, stopped with HClO_4_, and then neutralized with KOH. G6P was determined by mixing 50 *μ*L of sample with 200 *μ*L of 100 mM HEPES buffer (pH 7.0) containing 10 mM MgCl_2_, 1 mM NAD^+^, 1 U/mL *β*-PGM, and 1 U/mL G6PDH. After incubation at 37°C for 15 min, the increase of absorbance at 340 nm was measured. One enzyme unit was defined as the amount of enzyme that produced 1 *μ*mol of *β*-G1P from maltose per minute.

The activities of the rest of the enzymes were measured at 37°C using the method described previously, as listed in Table S2.

### 2.6. Electrochemical Measurement

All electrochemical experiments were conducted at 37°C using a CHI660E Potentiostat (CH Instruments Inc.). The enzymatic reaction solution contained 100 mM HEPES buffer (pH 7.3), 100 mM NaCl, 8 mM NAD^+^, 10 mM MgCl_2_, 0.5 mM MnCl_2_, 0.5 mM sodium hexametaphosphate, 0.5 mM thiamine pyrophosphate, 2 mM potassium phosphate, 100 mg/L ampicillin, 50 mg/L kanamycin, enzymes, and 0.5 mM maltose. For electrochemical measurements using a 10 mL three-electrode system, the working electrode, the reference electrode, and the counter electrode were prepared as previously described [11] and were immersed into the enzymatic reaction solution. For the determination of Faraday efficiency, chronoamperometry measurements at 0.16 V were performed to record the generated current as a function of the reaction time. The applied potential of 0.16 V for oxidation was determined by cyclic voltammetry (CV). Nitrogen flushing was used to remove oxygen in this three-electrode system. The total charge was calculated based on time and the measured current. The Faraday efficiency (ƞF) was calculated based on a previously described method [38]. Theoretically, 48 electrons can be generated from each maltose molecule. This value was used for the calculation of ƞF.

To evaluate the current and power densities, a mediated electron transfer (MET) type of two-chamber enzymatic fuel cell comprised of the cathode, bioanode, and a Nafion 212 membrane was constructed. A 1×1 cm^2^ carbon felt was placed in the middle of the cathode chamber containing ferricyanide solution and connected with a titanium wire. Another 1×1 cm^2^ carbon felt adsorbed with VK_3_ was placed in the middle of the anodic chamber containing the enzymatic reaction solution as described above and connected to an external circuit through the titanium wire. The volumes of the anode and cathode solutions in the container were both 4 mL. Linear sweep voltammetry at a scan rate of 1 mV/s was then conducted. The current density was calculated based on the surface area of the bioanode. The power density was calculated from the current density and the recorded potential.

### 2.7. Production of FDP from Maltose

Proof-of-concept production of FDP from 5 g/L (13.9 mM) maltose was carried out at 37°C in 100 mM HEPES buffer (pH 7.0) containing 5 mM MgCl_2_, 100 mg/L ampicillin, 50 mg/L kanamycin, 20 mM potassium phosphate, 60 mM sodium pyrophosphate, 8 mM sodium hexametaphosphate, and 3 U/mL of each enzyme. The reaction volume was 1 mL. Optimization of enzyme concentrations was performed under the same conditions as described above, except that the concentration of a single enzyme was varied while the concentrations of the other four enzymes were fixed. The reaction was carried out for 5 h. To compromise between high product titer and low cost of enzymes, the enzyme concentration that resulted in a relatively high FDP titer was chosen as the optimal value. This optimized enzyme concentration was then used for the subsequent enzyme optimization processes. Enzymes were optimized in the order of MP, *β*-PGM, PPGK, PGI, and PP_i_-PFK through this systematic titration method.

Fed-batch production of FDP was carried out at 37°C in 100 mM HEPES buffer (pH 7.0) containing 50 mM MgCl_2_, 100 mg/L ampicillin, 50 mg/L kanamycin, 20 mM potassium phosphate, 80 mM sodium hexametaphosphate, 5 U/mL MP, 20 U/mL *β*-PGM, 10 U/mL PPGK, 5 U/mL PGI, and 10 U/mL PP_i_-PFK. The reaction volume was 2 mL. At 0 h, a final concentration of 20 g/L (55.6 mM) maltose was added together with 224 mM sodium pyrophosphate to initiate the reaction. An aliquot of the reaction mixture was taken every hour to determine the concentration of FDP. When the reaction reached equilibrium, another 15 g/L (41.7 mM) maltose and 168 mM sodium pyrophosphate were supplemented to the system. This operation was repeated when the reaction reach equilibrium again.

### 2.8. Quantification of Maltose and FDP

Maltose was quantified by HPLC equipped with a refractive index detector. An Aminex HPX-87H column (Bio-Rad) was used for separation, and the column temperature was maintained at 80°C. 5 mM H_2_SO_4_ was used as mobile phase at a flow rate of 0.6 mL/min. The injection volume was set as 20 *μ*L. The concentrations of maltose in the samples were calculated based on the peak intensities with commercial maltose monohydrate (molecular weight: 360.31) as a reference. The quantification of FDP followed the method of Wang et al. [14]. 10 *μ*L of the sample was mixed with 300 *μ*L of FDP assay solution which contained 200 mM triethanolamine buffer (pH 7.6), 0.1 mM NADH, 0.135 U/mL ALD (purchased from Sigma-Aldrich), 2.5 U/mL TIM from *Thermus thermophilus*, and 0.4 U/mL glycerol 3-phosphate dehydrogenase (GPDH, purchased from Sigma-Aldrich). The reaction was conducted at 30°C for 30 min. The amount of FDP in the sample was calculated based on the consumption of NADH which could be measured at 340 nm by a spectrophotometer. Results were means±standard deviation of three parallel replicates.

## 3. Results

### 3.1. Stoichiometric Production of G6P from Maltose

**Figure 2:**
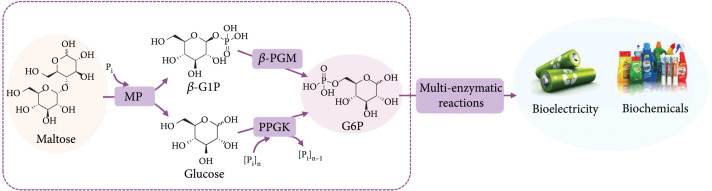
Schematic representation of the *in vitro* synthetic enzymatic reactions for the stoichiometric utilization of maltose. The three-enzyme module for the conversion of maltose to G6P was marked by a rectangular frame. P_i_: orthophosphate; [P_i_]_n_: polyphosphate. Enzymes and the other compounds are abbreviated as described in the text.

In this study, we designed an *in vitro* synthetic enzymatic reaction scheme to utilize maltose for biomanufacturing (Figure 2). The key of this scheme is a three-enzyme reaction module aiming at the stoichiometric conversion of maltose into G6P. In this module, (1) maltose is phosphorylated by maltose phosphorylase (MP) to yield *β*-G1P and glucose, (2) *β*-G1P requires *β*-phosphoglucomutase (*β*-PGM) for its conversion to G6P, and (3) glucose is phosphorylated by polyphosphate glucokinase (PPGK) to produce G6P. In this way, one maltose molecule theoretically yields two G6P molecules which can subsequently be used for the enzymatic biomanufacturing of biochemicals or bioelectricity.

**Figure 3:**
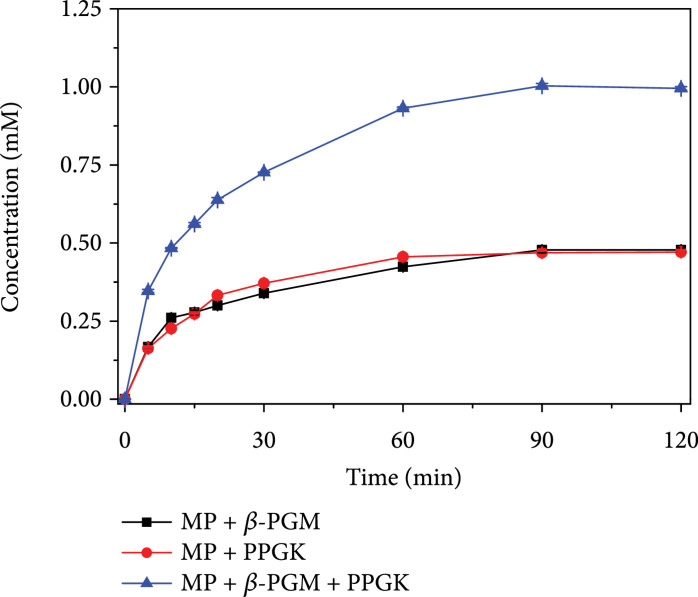
Production of G6P from maltose by the *in vitro* synthetic enzymatic reaction modules. The reaction was performed at 37°C in 100 mM HEPES buffer (pH 7.3) containing 100 mM NaCl, 2 mM potassium phosphate, 0.5 mM sodium hexametaphosphate, 10 mM MgCl_2_, 0.5 mM MnCl_2_, 1 U/mL MP, 1 U/mL *β*-PGM, and 1 U/mL PPGK.

To construct the three-enzyme maltose-to-G6P reaction module, a known MP from *B. subtilis*, a known engineered *T. fusca* PPGK, and a putative thermostable *β*-PGM from *P. horikoshii* OT3 (NCBI reference sequence: WP_010884842.1) were cloned, expressed, and purified. *β*-PGM was able to utilize *β*-G1P but showed no activity for *α*-G1P, thus classifying this enzyme as EC 5.4.2.6. *β*-PGM functioned optimally at around 70°C in HEPES buffer at pH 7.0 (Figure S1), with a specific activity of 34.7 U/mg. Meanwhile, it also had catalytic activity at 37°C. Because MP was from a mesophilic source, the *in vitro* reaction temperature was set as 37°C. At this temperature and pH 7.0, the specific activities of the purified MP, *β*-PGM, and PPGK were 7.0, 1.6, and 40.0 U/mg, respectively. Then, the three-enzyme *in vitro* reaction module was tested for its ability to generate G6P, with each enzyme loaded at 1 U/mL. When 0.5 mM maltose was used as the substrate, a double-enzyme module comprised of MP and *β*-PGM produced 0.48 mM G6P after 120 min of reaction, while the double-enzyme module comprised of MP and PPGK produced 0.47 mM G6P. In comparison, the three-enzyme module produced 1.0 mM G6P within 120 min (Figure 3), indicating the stoichiometric production of G6P from maltose as we expected.

### 3.2. Production of Bioelectricity from Maltose via G6P

**Figure 4:**
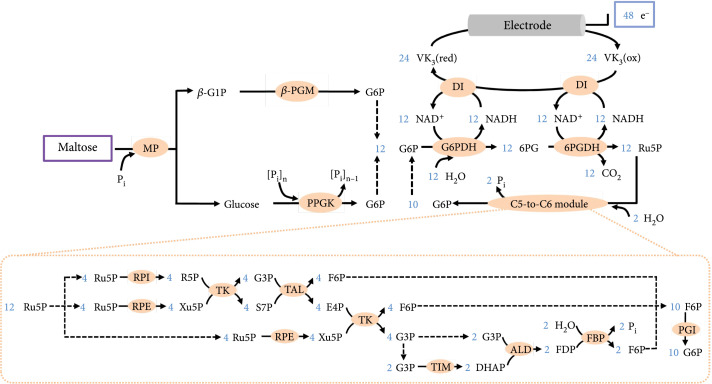
Pathway for the generation of bioelectricity from maltose by a 14-enzyme *in vitro* synthetic biobattery. P_i_: orthophosphate; [P_i_]_n_: polyphosphate; 6PG: 6-phosphogluconate; R5P: ribose 5-phosphate; Xu5P: xylulose 5-phosphate; G3P: glyceraldehyde 3-phosphate; S7P: sedoheptulose 7-phosphate; F6P: fructose 6-phosphate; E4P: erythrose 4-phosphate; DHAP: dihydroxyacetone 3-phosphate. Enzymes and the other compounds are abbreviated as described in the text.

After confirming that G6P could be stoichiometrically generated from maltose, this three-enzyme reaction module was extended for bioelectricity generation by adding more downstream enzymes. The reason for using bioelectricity generation to demonstrate the stoichiometric utilization of maltose is that the signal of generated electricity can be precisely captured by the instruments even at a low maltose concentration. In this designed biobattery, G6P produced from maltose was oxidized by two cascade enzymes, glucose 6-phosphate dehydrogenase (G6PDH) and 6-phosphogluconate dehydrogenase (6PGDH), generating CO_2_ and ribulose 5-phosphate (Ru5P). Accompanying with the oxidation of one G6P molecule, two NAD^+^ molecules were reduced to NADH. Subsequently, each NADH was reoxidized into NAD^+^ by diaphorase (DI) and produced two electrons which could be transferred to the anode via immobilized vitamin K_3_ (VK_3_). In this case, the oxidation of one maltose molecule could stoichiometrically produce 8 electrons by the 6-enzyme biobattery (Figure S2). Another 8 enzymes, which were ribose 5-phosphate isomerase (RPI), ribulose 5-phosphate 3-epimerase (RPE), transketolase (TK), transaldolase (TAL), triose-phosphate isomerase (TIM), fructose-bisphosphate aldolase (ALD), fructose bisphosphatase (FBP), and phosphoglucose isomerase (PGI), constituted a C5-to-C6 recycling module for the conversion of every Ru5P into 5/6 molecule of G6P (Figure 4). Theoretically, this 14-enzyme biobattery could generate 48 electrons from the complete oxidation of each maltose molecule.

**Figure 5:**
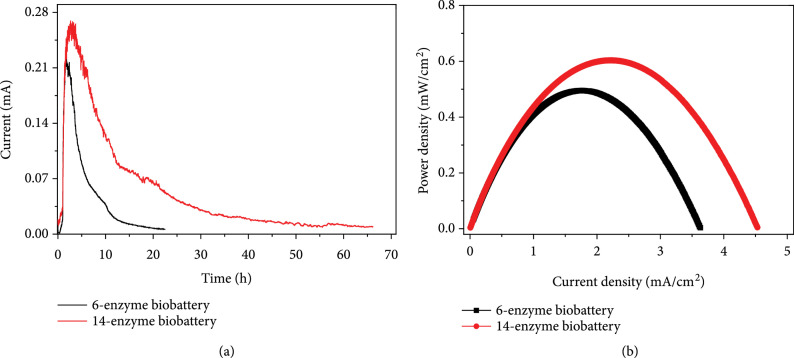
Bioelectricity generation from the oxidation of maltose at near-theoretical efficiency: (a) profiles of the current produced versus time; (b) profiles of power density versus current density. 3 U/mL MP, 3 U/mL *β*-PGM, 3 U/mL PPGK, 5 U/mL G6PDH, 5 U/mL 6PGDH, and 5 U/mL DI were used. For the 14-enzyme biobattery, the reaction solution also contained a C5-to-C6 recycling module comprised of RPI, RPE, TK, TAL, TIM, ALD, FBP, and PGI. Each of these C5-to-C6 enzymes was loaded at 1 U/mL.

All enzymes used in this biobattery were purified and analyzed by SDS-PAGE for their purities (Figure S3). We first tested the performance of the 6-enzyme biobattery via a proof-of-concept trial using 1 U/mL of MP, *β*-PGM, PPGK, and 5 U/mL of G6PDH, 6PGDH, and DI. After adding 0.5 mM maltose to initiate the reaction, a sharp increase of the current was observed (Figure S4). The current reached its peak of 0.09 mA at around 1.5 h and then declined over time. Within a reaction time of 20 h, the cumulative electric charges generated from 10 mL of the reaction mixture were 1.63 C, whereas the theoretical electric charges generated from 0.5 mM maltose would be 3.86 C. In this case, the Faraday efficiency was only 42.2%. Enhancing the loading concentrations of MP, *β*-PGM, and PPGK from 1 U/mL to 3 U/mL resulted in an enhancement of cumulative electric charges generated within 20 h to 3.85 C, corresponding to a near-theoretical Faraday efficiency of 99.7% (Figure 5(a), black curve). The maximum current density and power density of this 6-enzyme biobattery were 3.6 mA/cm^2^ and 0.5 mW/cm^2^, respectively (Figure 5(b), black curve). We then added the C5-to-C6 recycling module to construct a 14-enzyme biobattery. Within the reaction period of 65 h, the 14-enzyme biobattery produced 22.32 C of electricity, corresponding to a near-theoretical Faraday efficiency of 96.4% (Figure 5(a), red curve). Compared with the 6-enzyme system, the 14-enzyme system displayed enhancements of both maximum current density and power density, which were 4.5 mA/cm^2^ and 0.6 mW/cm^2^, respectively (Figure 5(b), red curve). These results suggested that the addition of the C5-to-C6 recycling module facilitated the complete oxidation of maltose and enhanced the discharge rate of the biobattery. In addition, the maximal power density of the 14-enzyme biobattery at 37°C was comparable with the performance of the maltodextrin-based biobattery (0.35 mW/cm^2^ at 23°C and 0.8 mW/cm^2^ at 50°C) [38] and was higher than the power density of another enzymatic biobattery using xylose as input (0.36 mW/cm^2^ at 37°C) [11].

### 3.3. Production of FDP from Maltose via G6P

**Figure 6:**
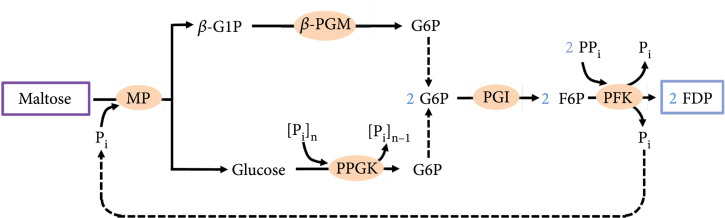
Pathway for the ATP-free synthesis of FDP from maltose by an *in vitro* synthetic biosystem. Enzymes and compounds are abbreviated as described in the text.

After confirming that the three-enzyme *in vitro* reaction module could achieve stoichiometric utilization of maltose, another *in vitro* biosystem was designed for the biomanufacturing of chemicals using relatively higher concentrations of maltose. Fructose 1,6-diphosphate (FDP) was chosen as the product for the following two reasons: (1) FDP plays a cytoprotective role in many pathological situations, making it a useful therapeutic agent for ischaemic injury [39] and a potential treatment agent in scenarios such as convulsions [40], UV-induced oxidative skin damage [41], and diabetic testicular complication [42]; (2) FDP is a high-energy chemical with two phosphate groups per module; therefore, the accumulation of phosphate ions in the *in vitro* biosystem for FDP production will be less severe than that in a similar system for phosphate-free products such as inositol. In this study, based on the three-enzyme maltose-to-G6P module, we designed an *in vitro* synthetic biosystem for the ATP-free synthesis of FDP from maltose. In addition to MP, *β*-PGM, and PPGK, this system also contained PGI which isomerized G6P into fructose 6-phosphate (F6P) and pyrophosphate-dependent phosphofructokinase (PP_i_-PFK) which phosphorylated F6P into FDP in the presence of pyrophosphate (PP_i_) (Figure 6). Half of the orthophosphate ions released by PP_i_-PFK could be reused by MP. Theoretically, each maltose molecule would be capable of the synthesis of two FDP molecules.

**Figure 7:**
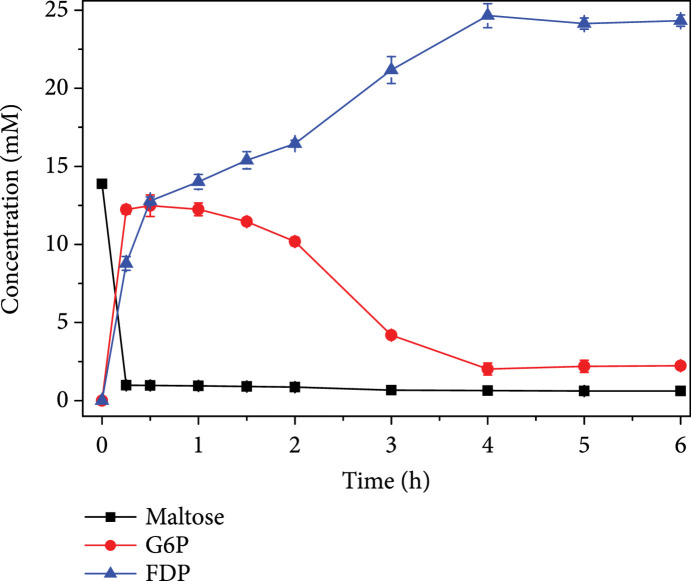
One-pot synthesis of FDP from maltose under optimized conditions. The reaction was performed at 37°C in 100 mM HEPES buffer (pH 7.0) containing 5 g/L (13.9 mM) maltose, 1 U/mL MP, 4 U/mL *β*-PGM, 2 U/mL PPGK, 1 U/mL PGI, 2 U/mL PP_i_-PFK, 5 mM MgCl_2_, 100 mg/L ampicillin, 50 mg/L kanamycin, 20 mM potassium phosphate, 60 mM sodium pyrophosphate, and 8 mM sodium hexametaphosphate.

All enzymes used in this FDP-producing biosystem were purified and analyzed by SDS-PAGE for their purities (Figure S5). We started with a proof-of-concept trial using 5 g/L (13.9 mM) maltose to produce FDP. Maltose was quantified by HPLC with commercial maltose monohydrate as a standard (Figure S6), while FDP was quantified by the enzymatic method. After reacting at 37°C, pH 7.0 for 15 min, most maltose in the sample was consumed (Figure S7a). When the reaction reached equilibrium, the residual maltose in the sample was only 0.63 mM, indicating that 95.5% of maltose had been consumed (Figure S7b). Meanwhile, there was 2.32 mM G6P remaining in the reaction mixture, and the FDP concentration was 21.73 mM, corresponding to 78.2% of the theoretical product yield and suggesting the production of 1.6 FDP molecules from each maltose. To improve the product yield, we first investigated the effect of pH on the reaction system and found that no further pH optimization was necessary because the system produced the highest amount of FDP at pH 7.0 (Figure S8). Next, we optimized the loading amounts of the five enzymes by systematic titration. The optimal concentrations of MP, *β*-PGM, PPGK, PGI, and PP_i_-PFK were 1, 4, 2, 1, and 2 U/mL, respectively (Figure S9). At the optimized enzyme concentrations, one-pot production of FDP from maltose by the *in vitro* biosystem containing MP, *β*-PGM, PPGK, PGI, and PP_i_-PFK was carried out again (Figure 7). The reaction reached equilibrium at 4 h when 0.64 mM maltose, 2.02 mM G6P, and 24.65 mM FDP were detected. It was observed that optimization of enzyme loading amounts facilitated the increase of FDP yield from 78.2% to 88.7% of the theoretical value. The consumption of each maltose molecule yielded around 1.9 FDP molecules, indicating the near-stoichiometric synthesis of FDP.

**Figure 8:**
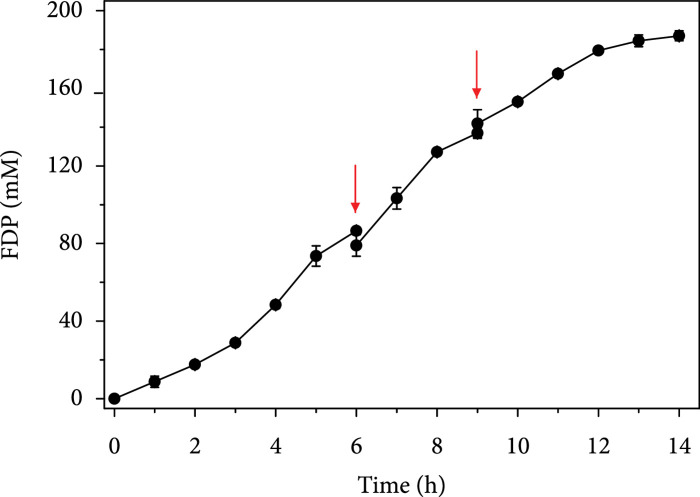
Fed-batch reaction for the synthesis of FDP from high concentrations of substrates. The reaction was carried out at 37°C in 100 mM HEPES buffer (pH 7.0) containing 50 mM MgCl_2_, 100 mg/L ampicillin, 50 mg/L kanamycin, 20 mM potassium phosphate, 80 mM sodium hexametaphosphate, 5 U/mL MP, 20 U/mL *β*-PGM, 10 U/mL PPGK, 5 U/mL PGI, and 10 U/mL PP_i_-PFK. At 0 h, a final concentration of 20 g/L (55.6 mM) maltose was added together with 224 mM sodium pyrophosphate to initiate the reaction. Red arrows indicated the time when another 15 g/L (41.7 mM) maltose and 168 mM sodium pyrophosphate were supplemented to the system.

To evaluate the industrialization potential of this system for FDP production, it is necessary to test a higher substrate concentration such as 50 g/L. We adopted a step-wise strategy for the addition of substrates which was necessary due to the limited solubility of pyrophosphate at 37°C. Besides, fed-batch addition of pyrophosphate was specifically required because high concentrations of inorganic phosphate could precipitate magnesium ions [14], which would in turn affect the activities of magnesium-dependent enzymes such as *β*-PGM, PPGK, PGI, and PP_i_-PFK. Meanwhile, maltose was added in a fed-batch manner to avoid the accumulation of glucose in the system which might inhibit the activity of MP. Considering that a portion of pyrophosphate might chelate with magnesium ions, pyrophosphate and maltose were added at a constant molar ratio of 4 : 1 which was higher than their stoichiometric molar ratio of 2 : 1. A high concentration of MgCl_2_ of 50 mM was also used to ensure the presence of sufficient magnesium ions in the reaction system. The loading amount of each enzyme was five times as the previously optimized concentration for 5 g/L maltose. This was a compromise strategy, because scaling up of enzyme loadings by tenfold would result in a total enzyme concentration of 47 g/L which might not only increase the viscosity of the reaction solution but also drastically raise the cost of enzymes. The reaction was carried out at 37°C, pH 7.0. At 0 h, 20 g/L (55.6 mM) maltose and 224 mM sodium pyrophosphate were added to initiate the reaction. After 6 h when the FDP titer reached approximately 86.5 mM, 15 g/L (41.7 mM) maltose and 168 mM pyrophosphate were supplemented to the system (Figure 8. The FDP titer kept increasing within the next 3 h to 137.0 mM. Then, another 15 g/L (41.7 mM) maltose and 168 mM pyrophosphate were added. At 14 h, the final titer of FDP was 187.0 mM, corresponding to an overall product yield of 67.3% of the theoretical value. Our system outperformed a previously reported *in vitro* synthetic biosystem which produced 125 mM FDP from 50 g/L maltodextrins [14].

## 4. Discussion

In this study, we chose maltose, the economic and most widely distributed *α*-linked glucose disaccharides in nature, as a substrate for *in vitro* biomanufacturing. A three-enzyme *in vitro* synthetic reaction module was constructed for the ATP-free, stoichiometric conversion of each maltose molecule to two G6P molecules. Then, downstream enzymes were added to this module to construct a 14-enzyme *in vitro* biobattery as well as a 5-enzyme FDP-producing *in vitro* biosystem. After system optimization, both the biobattery and the FDP-producing biosystem demonstrated near-theoretical performances. The 14-enzyme biobattery resulted in a Faraday efficiency of 96.4% and a maximal power density of 0.6 mW/cm^2^ at 37°C. This power density was comparable with the performance of a previously reported maltodextrin-based biobattery [38] and was higher than the power density of another enzymatic biobattery using xylose as input [11]. The 5-enzyme biosystem for FDP production also achieved a near-stoichiometric yield by producing 1.9 FDP molecules per maltose when 5 g/L of maltose was used as input. These results suggested the potential of the three-enzyme reaction module for the stoichiometric manufacturing of more value-added products from maltose via G6P, including biohydrogen, D-allulose, inositol, and glucosamine.

In common with several previously designed *in vitro* synthetic enzymatic biosystems using starch, cellulose, cellobiose, or sucrose as the substrate, the maltose-powered biosystems in this study start with the orthophosphate-dependent phosphorylation of substrate into G1P, followed by the isomerization of G1P to G6P. To construct a maltose-powered *in vitro* system, however, it is insufficient to merely replace the sugar substrate and the corresponding sugar phosphorylase of the established *in vitro* systems with maltose and MP. This is because the phosphorylation of starch, cellulose, cellobiose, and sucrose yielded *α*-G1P whereas the phosphorylation of maltose produced *β*-G1P. Unlike G6P and glucose which can undergo mutarotation to an equilibrium mixture of the *α* and *β* configurations due to their free anomeric carbons (C1), G1P has a phosphate group at its C1 position and hence is not able to spontaneously interconvert between its *α*- and *β*- forms. As a result, in addition to the utilization of MP, the *α*-PGMs (EC 5.4.2.2) which are specific for *α*-G1P in the previous *in vitro* systems should be replaced with a *β*-PGM (EC 5.4.2.6) for the conversion of maltose to G6P. Meanwhile, maltose and MP can be used for the *in vitro* enzymatic production of rare disaccharides such as kojibiose and nigerose which can be synthesized from *β*-G1P and glucose by the corresponding phosphorylases [43].

Our design of the biobattery was inspired by a previous study using maltodextrin for stoichiometric bioelectricity generation via G6P [38]. Therefore, we adopted the previously reported loading concentrations of the downstream enzymes (5 U/mL for G6PDH, 6PGDH, and DI) for our proof-of-concept test. However, a low Faraday efficiency of only 42.2% was obtained. Considering that the concentrations of upstream G6P-producing enzymes in the maltodextrin-based biobattery were also 5 U/mL, while MP, *β*-PGM, and PPGK in our system were loaded at only 1 U/mL, it was suspected that our low Faraday efficiency might be due to the low G6P-producing rate which did not match with the downstream bioelectricity generation process. Indeed, a simple system optimization by enhancing the loading concentration of each enzyme to 3 U/mL resulted in a near-theoretical Faraday efficiency. A main issue for further improvement of this biobattery is to prolong its lifetime. This may be achieved by methods such as enzyme immobilization [44] or the addition of BSA and Triton X-100 to the enzyme solution [38].

For the FDP-producing *in vitro* biosystem, optimization of enzyme concentrations resulted in an improvement of product yield from 78.2% to 88.7% of the theoretical value. The decrease of MP concentration, together with the increase of *β*-PGM concentration, might have mitigated the possible substrate inhibition of *β*-PGM, which was reported on the *E. coli* homologue [45]. Nevertheless, when increasing the input of maltose from 5 g/L to 50 g/L, the overall product yield dropped to 67.3% of the theoretical value despite a fed-batch strategy was adopted. Thermodynamic analysis using the eQuilibrator calculator [42] showed that the Gibbs free energy changes (*Δ*G) of reactions catalyzed by MP, *β*-PGM, PPGK, PGI, and PP_i_-PFK were 3.0, -7.4, -6.4, 2.5, and -0.6 kJ/mol, respectively, indicating the reaction catalyzed by PP_i_-PFK was reversible. Therefore, the accumulation of FDP at high concentrations might have caused PP_i_-PFK to cease to produce FDP. Another shortcoming of this FDP-producing system is the accumulation of inorganic phosphate during the reaction, as illustrated in Figure 6. This might inhibit the activities of magnesium-dependent enzymes through the chelation of phosphate with magnesium.

Several works can be done in the future to improve our current *in vitro* systems: (1) The MP used in this study was from *B. subtilis* and was mesophilic. Low thermostability of the enzymes in *in vitro* biosystems not only increases the production cost of enzymes but may also affect the long-term stability of the reaction system. The thermostability of MP thus needs to be improved, which can be achieved through protein engineering and the discovery of new thermostable MPs; (2) *β*-PGM from *P. horikoshii* has a low specific activity (1.6 U/mg) under the *in vitro* reaction conditions in this study, and hence, a relatively large amount of this enzyme was required for the optimal performance of the *in vitro* synthetic enzymatic biosystems. One solution to this issue is to increase the activity of *β*-PGM by protein engineering and gene mining. Another solution is to modify MP or to design novel enzymes to produce *α*-G1P through the phosphorylation of maltose, so that *α*-PGMs with high activities (such as the *α*-PGM from *Clostridium thermocellum* which is of around 885 U/mg at 37°C [46]) can be used instead of *β*-PGM. A careful mechanistic investigation on MP would benefit the latter idea. (3) The main drawback of the biosystems in this study is the accumulation of inorganic phosphates. To solve this problem, metal ions can be added during the reaction to chelate with the accumulated inorganic phosphates in the system. Another solution to mitigate the accumulation of phosphate is to replace the polyphosphate-dependent PPGK in our systems with some microorganisms to consume glucose for the production of single-cell proteins in parallel with the *in vitro* synthesis of biochemicals. (4) The present reaction pathway can be further extended for the high-yield synthesis of other downstream biochemicals such as 2-deoxy-ribose, allulose, tagatose, and ribulose [47].

In conclusion, we successfully designed and constructed an *in vitro* synthetic enzymatic reaction module which stoichiometrically produced two G6P molecules per maltose. Based on this reaction module, we constructed an enzymatic biobattery and an FDP-producing *in vitro* biosystem. The biobattery achieved a near-theoretical Faraday efficiency of 96.4% and a maximal power density of 0.6 mW/cm^2^. The FDP-producing *in vitro* biosystem generated 187.0 mM FDP from 50 g/L (139 mM) maltose through step-wise feeding of substrates. Our results provide a promising strategy for the complete utilization of maltose in both enzymatic fuel cells and *in vitro* biosynthesis of value-added chemicals in the future.

## Data Availability

All data is freely available upon request.

## Supplementary Materials

Supplementary Materials Figure S1: characterization of *β*-PGM. (a) Optimal temperature of *β*-PGM. (b) Optimal pH of *β*-PGM. Figure S2: pathway for the generation of bioelectricity from maltose by a 6-enzyme in vitro synthetic biobattery. Figure S3: SDS-PAGE analysis of the 14 recombinant enzymes for bioelectricity generation. Figure S4: proof-of-concept bioelectricity generation from maltose by the 6-enzyme biobattery. Figure S5: SDS-PAGE analysis of the 5 recombinant enzymes for FDP synthesis. Figure S6: HPLC analysis of commercial maltose as a standard. Figure S7: proof-of-concept production of FDP from maltose. Figure S8: optimization of reaction pH for FDP production. Figure S9: optimization of the enzyme loading amounts for FDP production. Table S1: the primers used in this study. Table S2: information of enzymes used in this study.

## References

[1] J. A. Rollin, T. K. Tam, and Y.-H. P. Zhang, “New biotechnology paradigm: cell-free biosystems for biomanufacturing,” *Green Chemistry*, vol. 15, no. 7, pp. 1708–1719, 2013

[2] Y.-H. P. Zhang, “Production of biocommodities and bioelectricity by cell-free synthetic enzymatic pathway biotransformations: challenges and opportunities,” *Biotechnology and Bioengineering*, vol. 105, no. 4, pp. 663–677, 2010 19998281 10.1002/bit.22630

[3] H. M. A. Moustafa, E. J. Kim, Z. G. Zhu, C. H. Wu, T. I. Zaghloul, M. W. W. Adams, and Y. H. P. Zhang, “Water splitting for high-yield hydrogen production energized by biomass xylooligosaccharides catalyzed by an enzyme cocktail,” *ChemCatChem*, vol. 8, no. 18, pp. 2898–2902, 2016

[4] C. You, T. Shi, Y. J. Li, P. P. Han, X. G. Zhou, and Y.-H. P. Zhang, “An *in vitro* synthetic biology platform for the industrial biomanufacturing of *myo*-inositol from starch,” *Biotechnology and Bioengineering*, vol. 114, no. 8, pp. 1855–1864, 2017 28409846 10.1002/bit.26314

[5] D. Meng, X. Wei, Y.-H. P. J. Zhang, Z. Zhu, C. You, and Y. Ma, “Stoichiometric conversion of cellulosic biomass by *in vitro* synthetic enzymatic biosystems for biomanufacturing,” *ACS Catalysis*, vol. 8, no. 10, pp. 9550–9559, 2018

[6] Y. Li, T. Shi, P. Han, and C. You, “Thermodynamics-driven production of value-added D-allulose from inexpensive starch by an *in vitro* enzymatic synthetic biosystem,” *ACS Catalysis*, vol. 11, no. 9, pp. 5088–5099, 2021

[7] S. Myung, J. Rollin, C. You, F. Sun, S. Chandrayan, M. W. Adams, and Y.-H. P. Zhang, “*In vitro* metabolic engineering of hydrogen production at theoretical yield from sucrose,” *Metabolic Engineering*, vol. 24, pp. 70–77, 2014 24836702 10.1016/j.ymben.2014.05.006

[8] H. Chen, R. Huang, E.-J. Kim, and Y.-H. P. J. Zhang, “Building a thermostable metabolon for facilitating coenzyme transport and *in vitro* hydrogen production at elevated temperature,” *ChemSusChem*, vol. 11, no. 18, pp. 3120–3130, 2018 30014617 10.1002/cssc.201801141

[9] E. J. Kim, J. E. Kim, and Y.-H. P. J. Zhang, “Ultra-rapid rates of water splitting for biohydrogen gas production through in vitro artificial enzymatic pathways,” *Energy & Environmental Science*, vol. 11, no. 8, pp. 2064–2072, 2018

[10] Z. Zhu, and Y.-H. P. Zhang, “In vitro metabolic engineering of bioelectricity generation by the complete oxidation of glucose,” *Metabolic Engineering*, vol. 39, pp. 110–116, 2017 27886975 10.1016/j.ymben.2016.11.002

[11] R. Wu, C. Ma, Y.-H. P. Zhang, and Z. Zhu, “Complete oxidation of xylose for bioelectricity generation by reconstructing a bacterial xylose utilization pathway *in vitro*,” *ChemCatChem*, vol. 10, no. 9, pp. 2030–2035, 2018

[12] P. Shi, R. Wu, J. Wang, C. Ma, Z. Li, and Z. Zhu, “Biomass sugar-powered enzymatic fuel cells based on a synthetic enzymatic pathway,” *Bioelectrochemistry*, vol. 144, article 108008, 2022 10.1016/j.bioelechem.2021.108008 34902664

[13] C. Zhong, C. You, P. Wei, and Y.-H. P. Zhang, “Thermal cycling cascade biocatalysis of *myo*-inositol synthesis from sucrose,” *ACS Catalysis*, vol. 7, no. 9, pp. 5992–5999, 2017

[14] W. Wang, M. Liu, C. You, Z. Li, and Y.-H. P. Zhang, “ATP-free biosynthesis of a high-energy phosphate metabolite fructose 1,6-diphosphate by in vitro metabolic engineering,” *Metabolic Engineering*, vol. 42, pp. 168–174, 2017 28624535 10.1016/j.ymben.2017.06.006

[15] D. Meng, X. Wei, X. Bai, W. Zhou, and C. You, “Artificial *in vitro* synthetic enzymatic biosystem for the one-pot sustainable biomanufacturing of glucosamine from starch and inorganic ammonia,” *ACS Catalysis*, vol. 10, no. 23, pp. 13809–13819, 2020

[16] P. H. Opgenorth, T. P. Korman, and J. U. Bowie, “A synthetic biochemistry module for production of bio-based chemicals from glucose,” *Nature Chemical Biology*, vol. 12, no. 6, pp. 393–395, 2016 27065234 10.1038/nchembio.2062

[17] T. P. Korman, P. H. Opgenorth, and J. U. Bowie, “A synthetic biochemistry platform for cell free production of monoterpenes from glucose,” *Nature Communications*, vol. 8, no. 1, p. 15526, 2017 10.1038/ncomms15526 PMC5458089 28537253

[18] B. S. Collins, C. T. Kelly, W. M. Fogarty, and E. M. Doyle, “The high maltose-producing alpha-amylase of the thermophilic actinomycete, *Thermomonospora curvata*,” *Applied Microbiology and Biotechnology*, vol. 39, no. 1, pp. 31–35, 1993 7763549 10.1007/BF00166844

[19] A. S. M. Noman, M. A. Hoque, P. K. Sen, and M. R. Karim, “Purification and some properties of *α*-amylase from post-harvest *Pachyrhizus erosus* L. tuber,” *Food Chemistry*, vol. 99, no. 3, pp. 444–449, 2006

[20] S. T. Sagu, E. J. Nso, T. Homann, C. Kapseu, and H. M. Rawel, “Extraction and purification of beta-amylase from stems of *Abrus precatorius* by three phase partitioning,” *Food Chemistry*, vol. 183, pp. 144–153, 2015 25863622 10.1016/j.foodchem.2015.03.028

[21] M. J. Pelczar Jr., and R. N. Doetsch, “On the direct fermentation of maltose,” *Science*, vol. 110, no. 2854, p. 256, 1949 17752428 10.1126/science.110.2854.256

[22] Y. Oda, and K. Tonomura, “Detection of maltose fermentation genes in the baking yeast strains of *Saccharomyces cerevisiae*,” *Letters in Applied Microbiology*, vol. 23, no. 4, pp. 266–268, 1996 8987701 10.1111/j.1472-765x.1996.tb00080.x

[23] W. Xia, W. Chen, W. Peng, and K. Li, “Industrial vitamin B12 production by *Pseudomonas denitrificans* using maltose syrup and corn steep liquor as the cost-effective fermentation substrates,” *Bioprocess and Biosystems Engineering*, vol. 38, no. 6, pp. 1065–1073, 2015 25561346 10.1007/s00449-014-1348-5

[24] Z. Zheng, Y. Xu, Y. Sun, W. Mei, and J. Ouyang, “Biocatalytic production of trehalose from maltose by using whole cells of permeabilized recombinant *Escherichia coli*,” *PLoS One*, vol. 10, no. 10, article e0140477, 2015 10.1371/journal.pone.0140477 PMC4603892 26462117

[25] N. Rashid, T. Kanai, H. Atomi, and T. Imanaka, “Among multiple phosphomannomutase gene orthologues, only one gene encodes a protein with phosphoglucomutase and phosphomannomutase activities in *Thermococcus kodakaraensis*,” *Journal of Bacteriology*, vol. 186, no. 18, pp. 6070–6076, 2004 15342576 10.1128/JB.186.18.6070-6076.2004 PMC515153

[26] Y. Wang, and Y.-H. P. Zhang, “A highly active phosphoglucomutase from *Clostridium thermocellum*: cloning, purification, characterization and enhanced thermostability,” *Journal of Applied Microbiology*, vol. 108, no. 1, pp. 39–46, 2010 19566723 10.1111/j.1365-2672.2009.04396.x

[27] W. J. Ray, and E. J. Peck, “12 Phosphomutases,” *The Enzymes*, P. D. Boyer, Ed., Academic Press, pp. 407–477, 1972

[28] C. You, X. Zhang, and Y.-H. P. Zhang, “Simple cloning via direct transformation of PCR product (DNA multimer) to *Escherichia coli* and *Bacillus subtilis*,” *Applied and Environmental Microbiology*, vol. 78, no. 5, pp. 1593–1595, 2012 22194286 10.1128/AEM.07105-11 PMC3294473

[29] W. Zhou, R. Huang, Z. Zhu, and Y.-H. P. J. Zhang, “Coevolution of both thermostability and activity of polyphosphate glucokinase from *Thermobifida fusca* YX,” *Applied and Environmental Microbiology*, vol. 84, no. 16, article e01224, 2018 10.1128/AEM.01224-18 PMC6070756 29884753

[30] R. Huang, H. Chen, W. Zhou, C. Ma, and Y. P. Zhang, “Engineering a thermostable highly active glucose 6-phosphate dehydrogenase and its application to hydrogen production *in vitro*,” *Applied Microbiology and Biotechnology*, vol. 102, no. 7, pp. 3203–3215, 2018 29480380 10.1007/s00253-018-8798-7

[31] Y. Wang, W. Huang, N. Sathitsuksanoh, Z. Zhu, and Y.-H. P. Zhang, “Biohydrogenation from biomass sugar mediated by *in vitro* synthetic enzymatic pathways,” *Chemistry & Biology*, vol. 18, no. 3, pp. 372–380, 2011 21439482 10.1016/j.chembiol.2010.12.019

[32] Z. Zhu, F. Sun, X. Zhang, and Y.-H. P. Zhang, “Deep oxidation of glucose in enzymatic fuel cells through a synthetic enzymatic pathway containing a cascade of two thermostable dehydrogenases,” *Biosensors & Bioelectronics*, vol. 36, no. 1, pp. 110–115, 2012 22521942 10.1016/j.bios.2012.04.001

[33] S. Huang, Y.-H. P. Zhang, and J. Zhong, “A thermostable recombinant transaldolase with high activity over a broad pH range,” *Applied Microbiology and Biotechnology*, vol. 93, no. 6, pp. 2403–2410, 2012 21947648 10.1007/s00253-011-3578-7

[34] F. Sun, X. Zhang, S. Myung, and Y. H. P. Zhang, “Thermophilic *Thermotoga maritima* ribose-5-phosphate isomerase RpiB: optimized heat treatment purification and basic characterization,” *Protein Expression and Purification*, vol. 82, no. 2, pp. 302–307, 2012 22333529 10.1016/j.pep.2012.01.017

[35] Y. R. Ding, R. S. Ronimus, and H. W. Morgan, “*Thermotoga maritima* phosphofructokinases: expression and characterization of two unique enzymes,” *Journal of Bacteriology*, vol. 183, no. 2, pp. 791–794, 2001 11133978 10.1128/JB.183.2.791-794.2001 PMC94940

[36] S. Myung, X. Zhang, and Y.-H. P. Zhang, “Ultra-stable phosphoglucose isomerase through immobilization of cellulose-binding module-tagged thermophilic enzyme on low-cost high-capacity cellulosic adsorbent,” *Biotechnology Progress*, vol. 27, no. 4, pp. 969–975, 2011 21630486 10.1002/btpr.606

[37] S. Myung, Y. Wang, and Y.-H. P. Zhang, “Fructose-1,6-bisphosphatase from a hyper-thermophilic bacterium *Thermotoga maritima*: characterization, metabolite stability, and its implications,” *Process Biochemistry*, vol. 45, no. 12, pp. 1882–1887, 2010

[38] Z. Zhu, T. K. Tam, F. Sun, C. You, and Y.-H. P. Zhang, “A high-energy-density sugar biobattery based on a synthetic enzymatic pathway,” *Nature Communications*, vol. 5, no. 1, p. 3026, 2014 10.1038/ncomms4026 24445859

[39] P. J. Marangos, A. W. Fox, B. J. Riedel, D. Royston, and Z. E. Dziewanowska, “Potential therapeutic applications of fructose-1,6-diphosphate,” *Expert Opinion on Investigational Drugs*, vol. 7, no. 4, pp. 615–623, 1998 15991998 10.1517/13543784.7.4.615

[40] X. Lian, F. A. Khan, and J. L. Stringer, “Fructose-1,6-bisphosphate has anticonvulsant activity in models of acute seizures in adult rats,” *Journal of Neuroscience*, vol. 27, no. 44, pp. 12007–12011, 2007 17978042 10.1523/JNEUROSCI.3163-07.2007 PMC6673383

[41] S. M. Ahn, J. S. Hwang, and S. H. Lee, “Fructose 1,6-diphosphate alleviates UV-induced oxidative skin damage in hairless mice,” *Biologial & Pharmaceutical Bulletin*, vol. 30, no. 4, pp. 692–697, 2007 10.1248/bpb.30.692 17409504

[42] M. Xu, D. Z. Dai, Q. Zhang, Y. S. Cheng, and Y. Dai, “Upregulated NADPH oxidase contributes to diabetic testicular complication and is relieved by strontium fructose 1,6-diphosphate,” *Experimental and Clinical Endocrinology & Diabetes*, vol. 118, no. 7, pp. 459–465, 2010 20200810 10.1055/s-0030-1248325

[43] G. Pergolizzi, S. Kuhaudomlarp, E. Kalita, and R. A. Field, “Glycan phosphorylases in multi-enzyme synthetic processes,” *Protein and Peptide Letters*, vol. 24, no. 8, pp. 696–709, 2017 28799504 10.2174/0929866524666170811125109 PMC5688430

[44] X. Xiao, H. Xia, R. Wu, L. Bai, L. Yan, E. Magner, S. Cosnier, E. Lojou, Z. Zhu, and A. Liu, “Tackling the challenges of enzymatic (bio)fuel cells,” *Chemical Reviews*, vol. 119, no. 16, pp. 9509–9558, 2019 31243999 10.1021/acs.chemrev.9b00115

[45] K. Mukherjee, T. Narindoshvili, and F. M. Raushel, “Discovery of a kojibiose phosphorylase in *Escherichia coli* K-12,” *Biochemistry*, vol. 57, no. 19, pp. 2857–2867, 2018 29684280 10.1021/acs.biochem.8b00392 PMC5953851

[46] X. Wei, Q. Li, C. Hu, and C. You, “An ATP-free *in vitro* synthetic enzymatic biosystem facilitating one-pot stoichiometric conversion of starch to mannitol,” *Applied Microbiology and Biotechnology*, vol. 105, no. 5, pp. 1913–1924, 2021 33544214 10.1007/s00253-021-11154-9

[47] W. Wang, J. Yang, Y. Sun, Z. Li, and C. You, “Artificial ATP-free *in vitro* synthetic enzymatic biosystems facilitate aldolase-mediated C-C bond formation for biomanufacturing,” *ACS Catalysis*, vol. 10, no. 2, pp. 1264–1271, 2020

