# Supplementary material for: Stoichiometric Conversion of Maltose for Biomanufacturing by In Vitro Synthetic Enzymatic Biosystems
Source: Biodes Res. 2022 Jul 1;2022:9806749. doi: 10.34133/2022/9806749 (PMC10521662; doi:10.34133/2022/9806749)
Supplement: Supplementary Materials — Figure S1: characterization of β-PGM. (a) Optimal temperature of β-PGM. (b) Optimal pH of β-PGM. Figure S2: pathway for the generation of bioelectricity from maltose by a 6-enzyme in vitro synthetic biobattery. Figure S3: SDS-PAGE analysis of the 14 recombinant enzymes for bioelectricity generation. Figure S4: proof-of-concept bioelectricity generation from maltose by the 6-enzyme biobattery. Figure S5: SDS-PAGE analysis of the 5 recombinant enzymes for FDP synthesis. Figure S6: HPLC analysis of commercial maltose as a standard. Figure S7: proof-of-concept production of FDP from maltose. Figure S8: optimization of reaction pH for FDP production. Figure S9: optimization of the enzyme loading amounts for FDP production. Table S1: the primers used in this study. Table S2: information of enzymes used in this study. [file 9806749.f1.docx]

Supplementary Materials

Supplementary figures


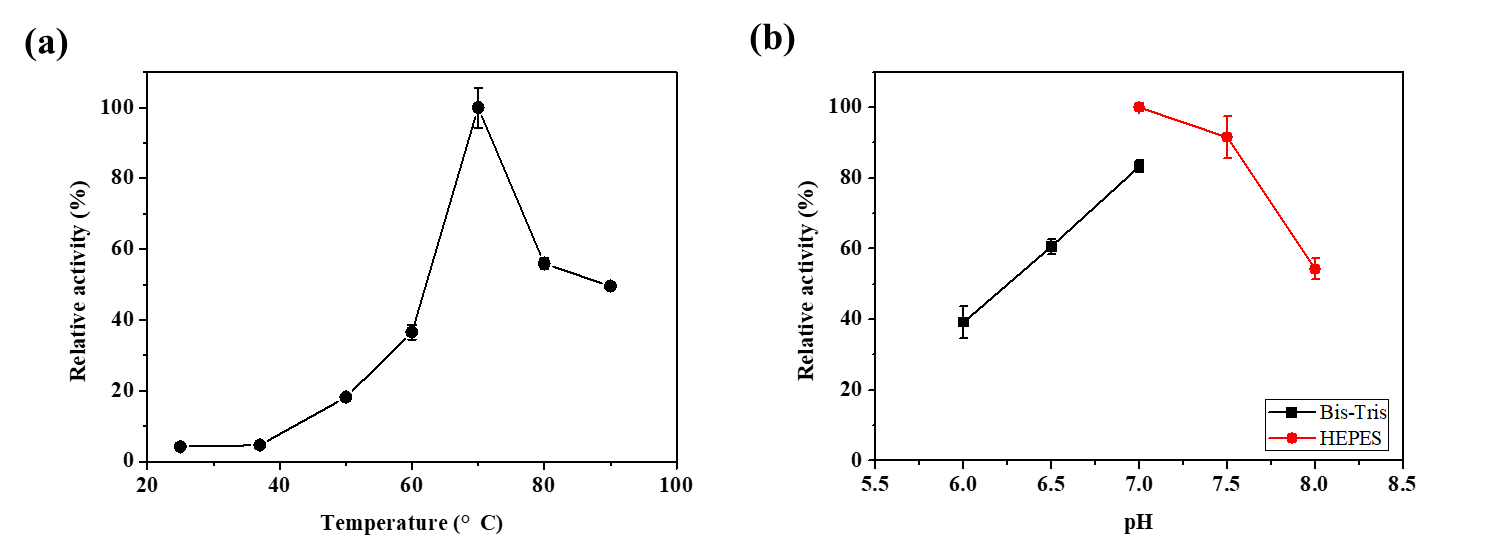


**FIGURE S1: Characterization of β-PGM.** (a) Optimal temperature of β-PGM. (b) Optimal pH of β-PGM.


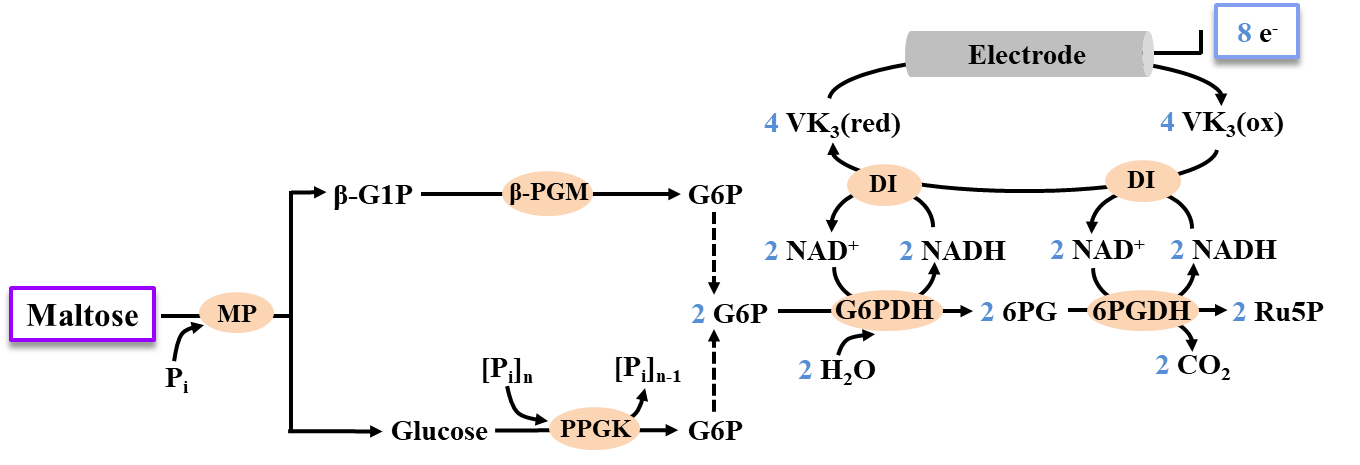


**FIGURE S2: Pathway for the generation of bioelectricity from maltose by a 6-enzyme *in vitro* synthetic biobattery.**


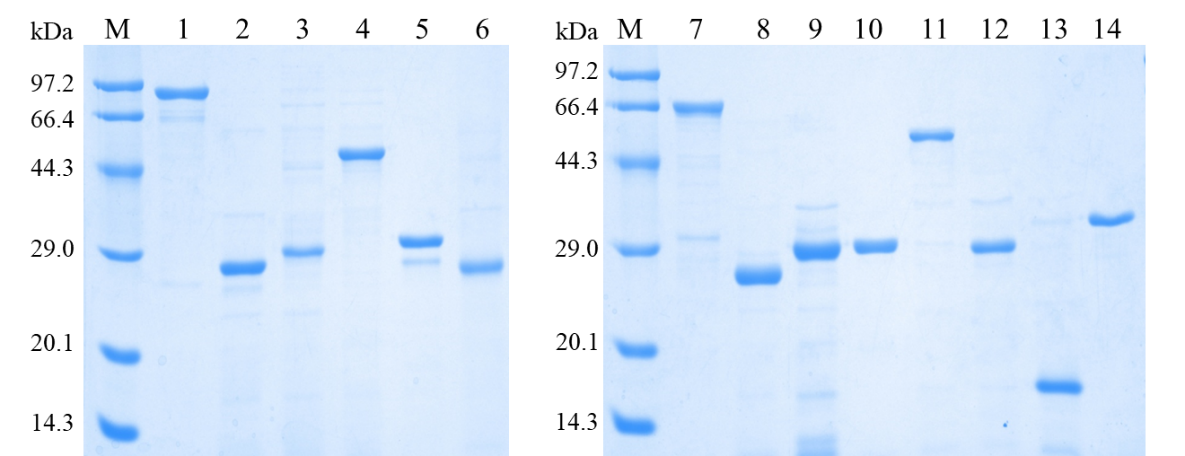


**FIGURE S3: SDS-PAGE analysis of the 14 recombinant enzymes for bioelectricity generation.** Detailed information of these enzymes was listed in Table S2. M, protein marker; lane 1, MP; lane 2, β-PGM; lane 3, PPGK; lane 4, G6PDH; lane 5, 6PGDH; lane 6, DI; lane 7, TK; lane 8, TAL; lane 9, TIM; lane10, FBP; lane 11, PGI; lane 12, RPE; lane 13, RPI; lane 14, ALD.

**
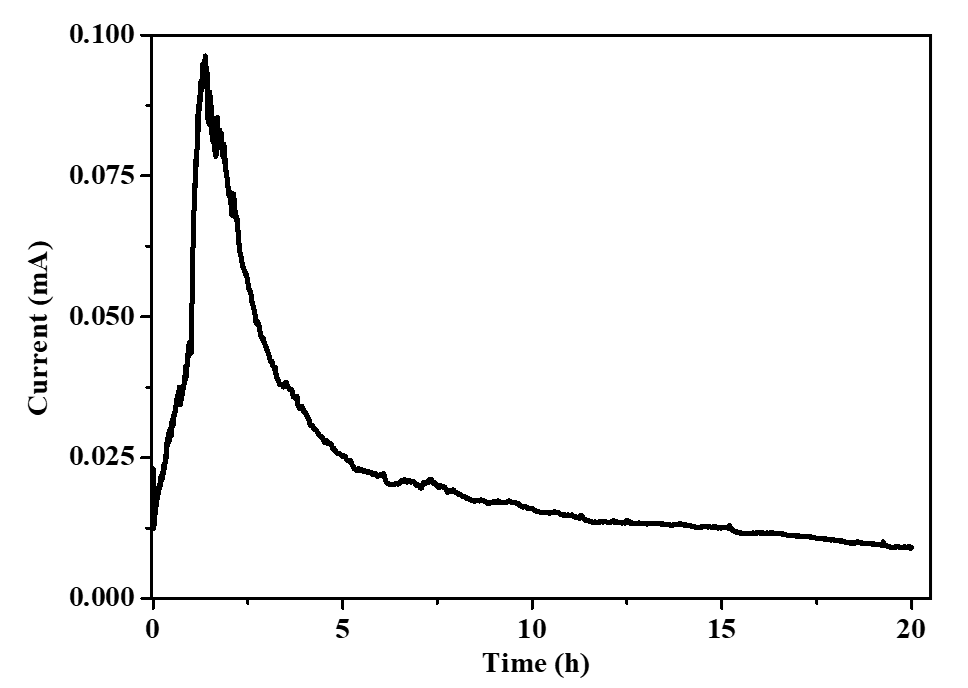
**

**FIGURE S4: Proof-of-concept bioelectricity generation from maltose by the 6-enzyme biobattery.** The experiment was performed using a 10-mL three-electrode system. 1 U/mL MP, 1 U/mL β-PGM, 1 U/mL PPGK, 5 U/mL G6PDH, 5 U/mL 6PGDH, and 5 U/mL DI were used.


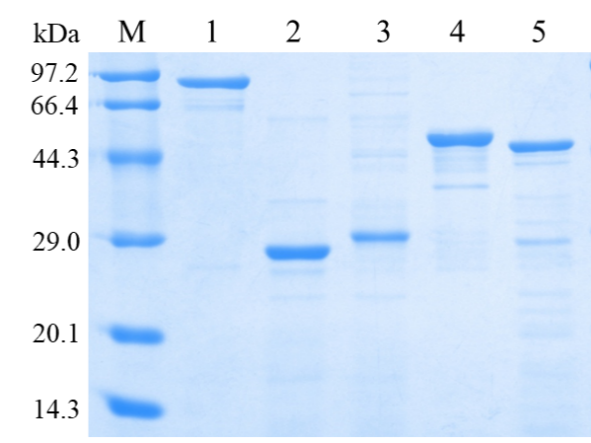


**FIGURE S5: SDS-PAGE analysis of the 5 recombinant enzymes for FDP synthesis.** Detailed information of these enzymes was listed in Table S2. M, protein marker; lane 1, MP; lane 2, β-PGM; lane 3, PPGK; lane 4, PGI; lane 5, PFK.


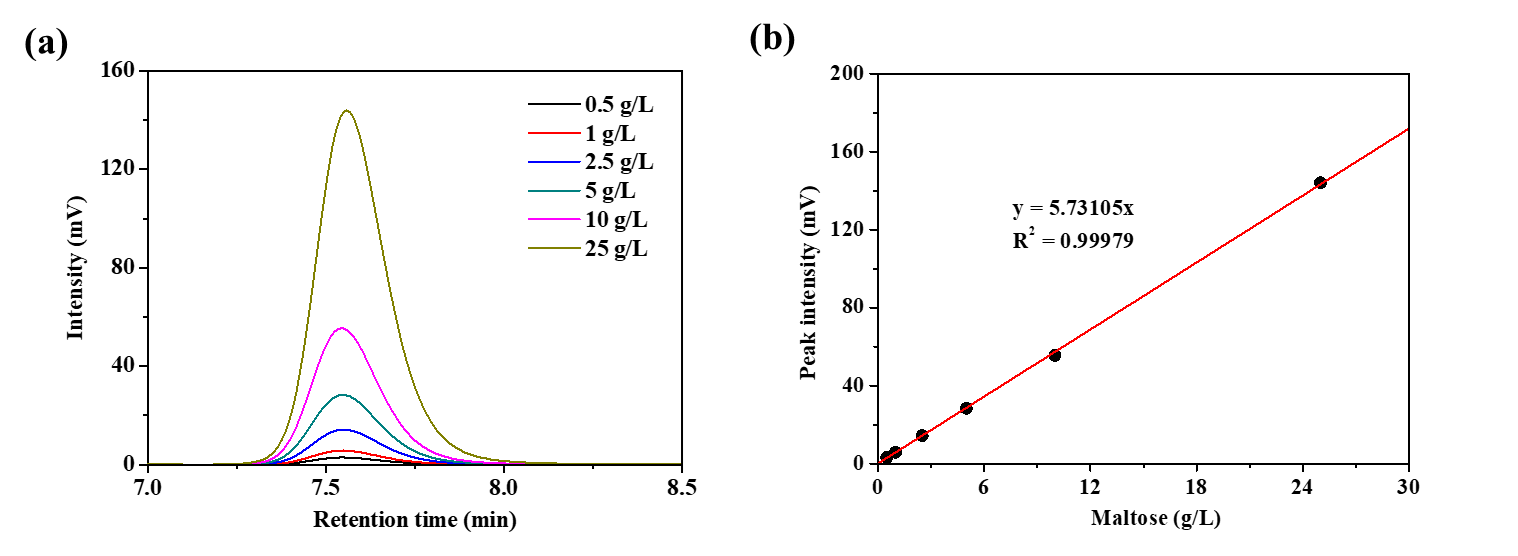


**FIGURE S6: HPLC analysis of commercial maltose as a standard.** (a) HPLC chromatograms of commercial maltose. (b) A standard curve of maltose. Maltose monohydrate with a molecular weight of 360.31 was used.


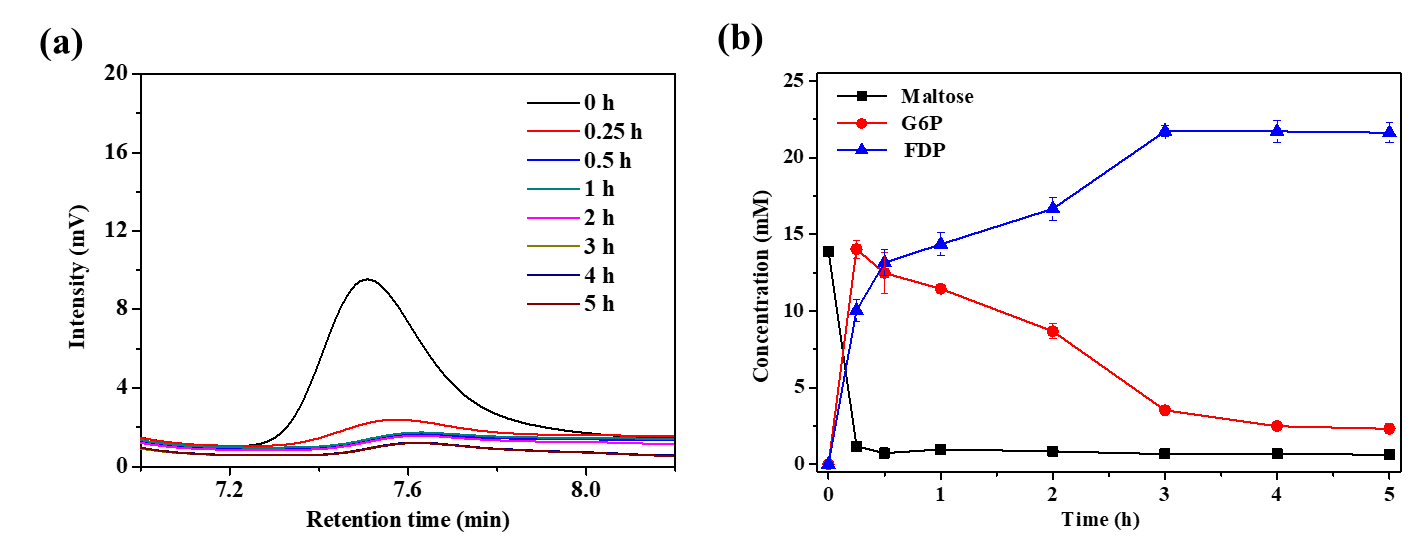


**FIGURE S7: Proof-of-concept production of FDP from maltose.** (a) HPLC chromatograms of maltose in the samples. (b) Time profiles of maltose, G6P, and FDP in the samples.


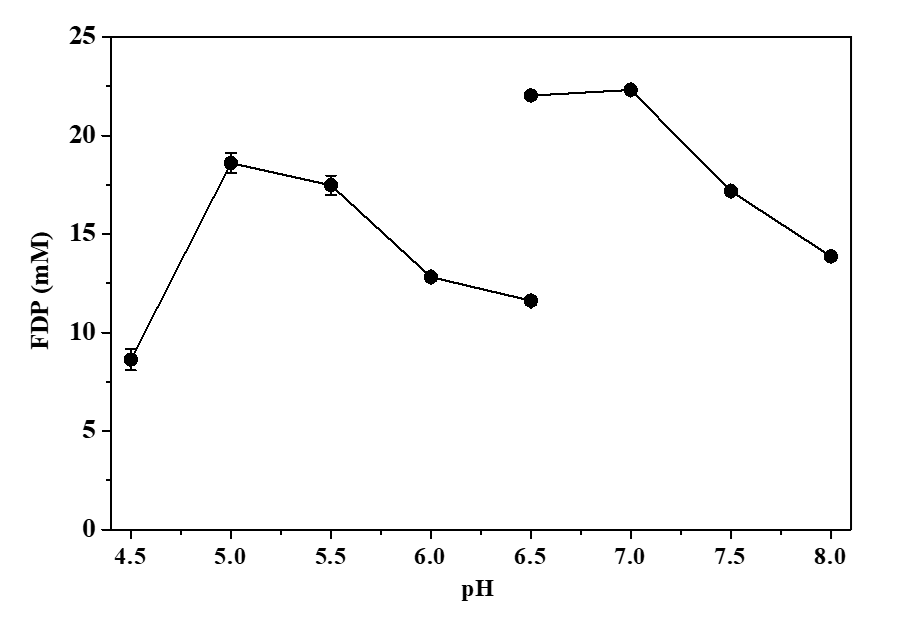


**FIGURE S8: Optimization of reaction pH for FDP production.** Reactions were conducted for 5 h in either 100 mM citric acid-sodium citrate buffer (pH 4.5–6.5) or 100 mM HEPES buffer (pH 6.5–8.0). The rest of reaction conditions were the same as those for the proof-of-concept experiment.


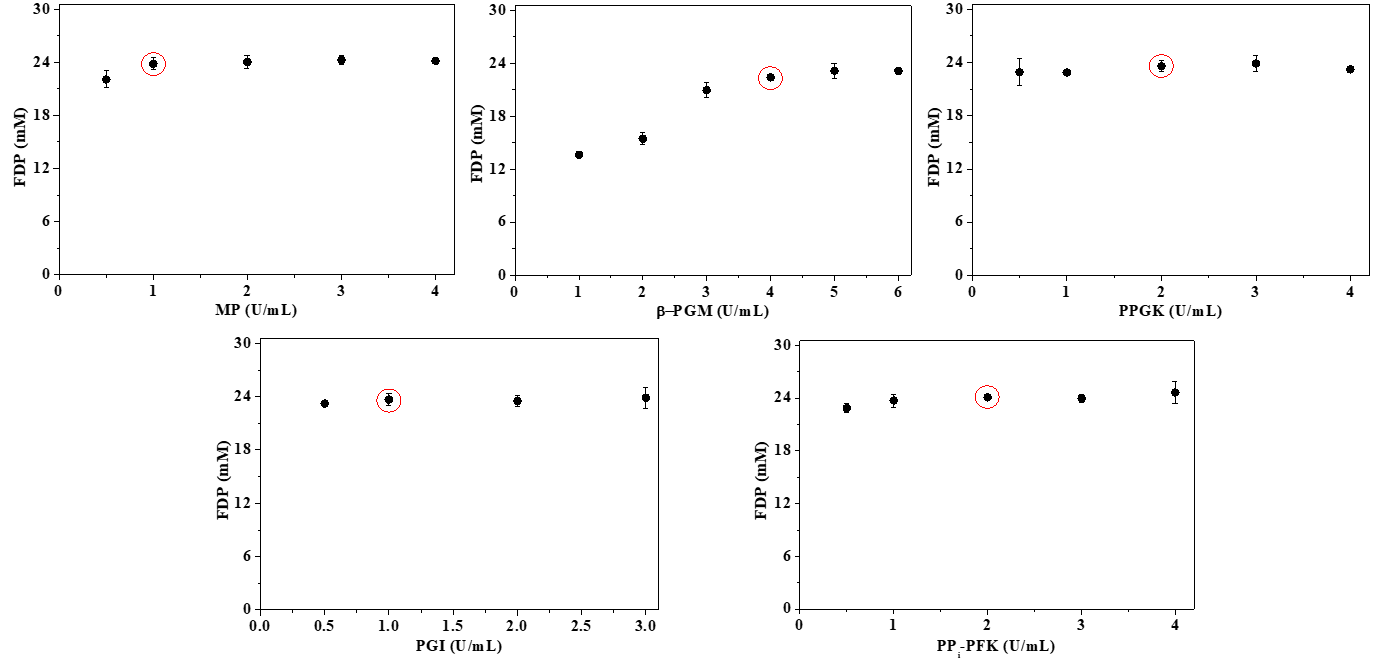


**FIGURE S9: Optimization of the enzyme loading amounts for FDP production.** Enzyme loading amounts were optimized sequentially by systematic titration in the order of MP, β-PGM, PPGK, PGI, and PFK. The rest of reaction conditions were the same as those for the proof-of-concept experiment. After 5 h of reaction, the amount of FDP in the samples were quantified. The optimal concentration for each enzyme was circled in the figure.

Supplementary tables

**TABLE S1: The primers used in this study.**

| Primers | Sequence (5’ → 3’) | Plasmid |
| --- | --- | --- |
| mp-IF | CTTTAAGAAGGAGATATACATATGATAAATCAGCGGTTATTTG | pET20b-*mp* |
| mp-IR | GGTGGTGGTGGTGCTCGAGCTCATTAGGCGTTCTCCTTTC |  |
| mp-VF | GAAAGGAGAACGCCTAATGAGCTCGAGCACCACCACCACC |  |
| mp-VR | CAAATAACCGCTGATTTATCATATGTATATCTCCTTCTTAAAG |  |
| βpgm-IF | CTTTAAGAAGGAGATATACATATGAAGCCGGCCTTAATATGGGAT | pET20b-*βpgm* |
| βpgm-IR | GTGGTGGTGGTGCTCGAGTATCTTCCCCTCCTTACATAGTTCGAATATG |  |
| βpgm-VF | CATATTCGAACTATGTAAGGAGGGGAAGATACTCGAGCACCACCACCAC |  |
| βpgm-VR | ATCCCATATTAAGGCCGGCTTCATATGTATATCTCCTTCTTAAAG |  |
| rpe-IF | GTTTAACTTTAAGAAGGAGATATACATATGGTGAAAATAGCAGCTTCAATTC | pET20b-*rpe* |
| rpe-IR | GTGGTGGTGGTGGTGGTGCTCGAGATCAGCAAATTCCTCTCTTTCCTG |  |
| rpe-VF | CAGGAAAGAGAGGAATTTGCTGATCTCGAGCACCACCACCACCACCAC |  |
| rpe-VR | GAATTGAAGCTGCTATTTTCACCATATGTATATCTCCTTCTTAAAGTTAAAC |  |

**TABLE S2: Information of enzymes used in this study.**

| Enzyme | EC number | Source organism | Purification method | Specific activity at 37 ℃ (U/mg) | Reference |
| --- | --- | --- | --- | --- | --- |
| Maltose phosphorylase (MP) | 2.4.1.8 | *Bacillus subtilis* | Ni-affinity chromatography | 7 | This study |
| Beta-phosphoglucomutase (β-PGM) | 5.4.2.6 | *Pyrococcus horikoshii* OT3 | Heat treatment | 1.6 | This study |
| Polyphosphate glucokinase (PPGK) mutant 4-1 | 2.7.1.63 | *Thermobifida fusca* YX | Heat treatment | 40 | [29] |
| Glucose 6-phosphate dehydrogenase (G6PDH) mutant 4-1 | 1.1.1.49 | *Zymomonas mobilis* | Ni-affinity chromatography | 200 | [30] |
| 6-Phosphogluconate dehydrogenase (6PGDH) | 1.1.1.44 | *Moorella thermoacetica* | Ni-affinity chromatography | 1.8 | [31] |
| Diaphorase (DI) | 1.6.99.3 | *Geobacillus stearothermophilus* | Ni-affinity chromatography | 600 | [32] |
| Transketolase (TK) | 2.2.1.1 | *Thermus thermophilus* HB8 | Heat treatment | 1.1 | [7] |
| Transaldolase (TAL) | 2.2.1.2 | *Thermotoga maritima* MSB8 | Heat treatment | 13 | [33] |
| Triose phosphate isomerase (TIM) | 5.3.1.1 | *T. thermophilus* HB8 | Heat treatment | 180 | [31] |
| Fructose bisphosphatase (FBP) | 3.1.3.11 | *T. maritima* MSB8 | CBM/intein | 6.0 | [37] |
| Phosphoglucose isomerase (PGI) | 5.3.1.9 | *Clostridium thermocellum* | CBM/intein | 400 | [36] |
| Ribulose 5-phosphate 3-epimerase (RPE) | 5.1.3.1 | *T. maritima* MSB8 | Heat treatment | 251 | This study |
| Ribose 5-phosphate isomerase (RPI) | 5.3.1.6 | *T. maritima* MSB8 | Heat treatment | 190 | [34] |
| Fructose-bisphosphate aldolase (ALD) | 4.1.2.13 | *T. thermophilus* HB8 | Heat treatment | 7.3 | [7] |
| Pyrophosphate phosphofructokinase (PP_i_-PFK) | 2.7.1.90 | *T. maritima* MSB8 | Heat treatment | 1.0 | [35] |
